# Association of Disease-Modifying Therapies with COVID-19 Susceptibility and Severity in Patients with Multiple Sclerosis: A Systematic Review and Network Meta-Analysis

**DOI:** 10.1155/2022/9388813

**Published:** 2022-09-21

**Authors:** Mahdi Barzegar, Shakiba Houshi, Erfan Sadeghi, Mozhgan Sadat Hashemi, Ghasem Pishgahi, Sara Bagherieh, Alireza Afshari-Safavi, Omid Mirmosayyeb, Vahid Shaygannejad, Aram Zabeti

**Affiliations:** ^1^Isfahan Neurosciences Research Center, Isfahan University of Medical Sciences, Isfahan, Iran; ^2^Department of Neurology, School of Medicine, Isfahan University of Medical Sciences, Isfahan, Iran; ^3^Department of Biostatistics and Epidemiology, Faculty of Health, Isfahan University of Medical Sciences, Isfahan, Iran; ^4^Students' Scientific Research Center, Tehran University of Medical Sciences, Tehran, Iran; ^5^Department of Biostatistics and Epidemiology, Faculty of Health, North Khorasan University of Medical Sciences, Bojnurd, Iran; ^6^Department of Neurology and Rehabilitation Medicine, Waddell center in Multiple Sclerosis, University of Cincinnati, Cincinnati, OH, USA

## Abstract

**Background:**

We conducted this study to assess the effect of disease-modifying therapies (DMTs) on coronavirus disease (COVID-19) susceptibility and severity in people with multiple sclerosis (MS).

**Methods:**

Available studies from PubMed, Scopus, EMBASE, Web of Science, and gray literature, including reference lists and conference abstracts, were searched from December 1, 2019, to July 26, 2021. We included cross-sectional, case-control, and cohort studies assessing the association of DMTs with risk of contracting COVID-19 or its outcomes in MS patients on univariate or multivariate regression analyses. We conducted a network meta-analysis (NMA) to compare the risk of COVID-19 and developing severe infection across DMTs.

**Results:**

Out of the initial 3893 records and 1883 conference abstracts, a total of 10 studies were included. Pairwise comparisons showed that none of the DMTs meaningfully affect the risk of acquiring infection. There was significant total heterogeneity and inconsistency across this NMA. In comparison with no DMT, dimethyl fumarate (0.62 (0.42, 0.93)), fingolimod (0.55 (0.32, 0.94)), natalizumab (0.50 (0.31, 0.81)), and interferon (0.42 (0.22, 0.79)) were associated with a decreased risk of severe COVID-19; but, rituximab was observed to increase the risk (1.94 (1.20, 3.12)). Compared to rituximab or ocrelizumab, all DMTs were associated with a decreased risk. Pairwise comparisons showed no differences across other DMTs. Interferon and rituximab were associated with the lowest and highest risks of severe COVID-19.

**Conclusion:**

Our study showed an increased risk of severe COVID-19 in patients on rituximab and ocrelizumab. No association with COVID-19 severity across other DMTs was observed.

## 1. Introduction

The outbreak of Coronavirus Disease-2019 (COVID-19), caused by severe acute respiratory syndrome coronavirus 2 (SARS-CoV-2), has led to a newly emerging pandemic. This globally spreading virus affects people in different ways, with manifestations ranging from no symptoms to hospitalization and death due to acute respiratory distress syndrome (ARDS) [1, 2]. More than one year after the outbreak of COVID-19, the number of reported COVID-19 cases exceeds 150 million, with more than 3.5 million deaths [3].

Multiple sclerosis (MS) is one of the most common demyelinating diseases in the central nervous system (CNS), affecting generally young female adults. MS patients often receive immunosuppressive agents which put those at greater risk of developing viral and bacterial infections [4–6]. This raised a question regarding whether people living with MS were at higher risk of COVID-19 and were more likely to develop severe symptoms when infected than the general population. A recent systematic review has suggested a mortality rate of 3.5% among MS patients considering suspected/confirmed COVID-19 cases, which is slightly higher than the rate of 2.2% among the general population [2, 7]. This study showed that patients on anti-CD20 agents had highest rates of hospitalization and mortality than those on other DMTs. Moreover, studies suggested an increased risk of developing the infection in MS patients on anti-CD20 agents [8, 9].

Knowledge of the association between disease-modifying therapies (DMTs) and COVID-19 susceptibility/severity is necessary to provide the best care for patients during the pandemic and could be important for policymakers to adopt vaccine strategies. However, the current evidence is inconsistent and unclear. Therefore, this study was conducted to present the current evidence regarding the effect of DMTs on COVID-19 susceptibility and severity in people living with MS.

## 2. Method

### 2.1. Inclusion and Exclusion Criteria

Studies were included according to the following criteria: population (participants), outcomes, and study types. The population (participants) consists of suspected or confirmed COVID-19 patients with a previous diagnosis of MS. Outcomes are the association of each specific DMT with COVID-19 susceptibility and outcomes reported based on univariate or multivariate regression analyses. The included study types are cross-sectional, case-control, and cohort studies. Studies with the following characteristics were excluded: (a) studies did not compare DMTs with each other; (b) studies pooled DMTs based on the mechanism of action (immune cell depleting medications or immune-cell trafficking inhibitors) or risk of systemic infection (no risk, mild, risk, or high risk); (c) nonpeer-reviewed articles; (d) non-English studies; (e) review articles and systematic review; and (f) qualitative studies.

### 2.2. Information Source and Search Strategy

We comprehensively searched electronic databases including PubMed, Scopus, EMBASE, and Web of Science from December 1, 2019, to July 26, 2021. The following search words were adapted: ((coronavirus OR Wuhan coronavirus OR novel coronavirus OR coronavirus disease OR COVID-19 OR 2019 novel coronavirus infection OR 2019-nCOV OR severe acute respiratory syndrome coronavirus 2 OR SARS-CoV-2) AND (Multiple Sclerosis OR (Sclerosis, Multiple) OR (Sclerosis, Disseminated) OR Disseminated Sclerosis OR (Multiple Sclerosis, Acute Fulminating)). We also screened the reference lists of identified articles, review studies, or other relevant documents for inclusion in the study. In addition, we also searched the online library and abstracts of the following congresses: 8^th^ American and European Committees for Treatment and Research in Multiple Sclerosis (ACTRIMS-ECTRIMS 2020), 145^th^ Annual Meeting American Neurological Association, Annual meeting America Academy of Neurology 2021, and 6^th^ Congress of the European Academy of Neurology, and to identify eligible studies that have not been published. We conducted this systematic review following the Preferred Reporting Items for Systematic Reviews and Meta-Analyses (PRISMA) guidelines [10].

### 2.3. Study Selection

Two researchers (MB and SB) independently screened the titles and abstracts of retrieved studies to identify the eligible studies. Then, the full text of the potentially eligible studies was reviewed. Disagreement regarding the study selection was resolved by consulting with a third investigator (AAS).

### 2.4. Quality Assessment

Two reviewers (OM and MB) independently evaluated the quality of the included studies using the Newcastle-Ottawa scale (NOS) quality tests [11]. Different checklists were used based on the study design. The third investigator solved any discrepancies (AAS) in quality assessment. We rated the quality of included studies by giving stars to three parameters of selection, comparability, and outcome according to the NOS guidelines (Supplementary file (available here)). Cross-sectional studies were categorized to very good, good, satisfactory, and unsatisfactory quality. Cohort studies were categorized as good, fair, and poor quality.

### 2.5. Data Extraction

Two researchers (MSH and GP) independently carried out the extraction of data. The following information was extracted from each eligible publication: first author's name, initial publication date, location of study, scenario of study, type of study, total number of MS patients, number of MS patients with confirmed/suspected COVID-19, and odds ratios (ORs) and their confidence intervals (95% CIs) of association between following DMTs and COVID-19 susceptibility or severity: interferon (IFN), glatiramer acetate (GA), dimethyl fumarate (DMF), teriflunomide (TRF), fingolimod (FNG), natalizumab (NTZ), rituximab (RTX), ocrelizumab (OCR), cladribine (CLA), and no DMT.

### 2.6. Data Synthesis

We conducted a network meta-analysis (NMA) on the risk of developing COVID-19 and its severity to assess the relative impacts of various DMTs. Model heterogeneity was estimated by *I*-square (*I*^2^) and tau-squared (*τ*^2^). The *Q* statistic (*Q*_total_) was decomposed to assess the heterogeneity (within study designs (*Q*_within_)) and inconsistency (between study designs (*Q*_between_)). League table was utilized to indicate all direct and indirect pairwise comparisons using ORs and their 95% CIs. ORs less than 1 indicated that the DMT reduced the risk of COVID-19 susceptibility or severity relative to the comparator DMT. A *P* score and net rank plot were also applied for ranking all DMTs based on their network estimates. A higher *P* score indicates a greater risk of COVID-19 susceptibility or severity. We did not perform sensitivity analysis based on the quality of studies since small number of included papers. However, only one included study had unsatisfied quality. We performed no publication bias test since less than 10 studies were included in each NMA [12]. The data were analyzed in Stata 14 software (Stata Corporation, College Station, Texas, USA) and R software (version 4.0.2, R Foundation for Statistical Computing, Vienna, Austria) using *netmeta* package.

## 3. Results

### 3.1. Study Selection

**Figure 1:**
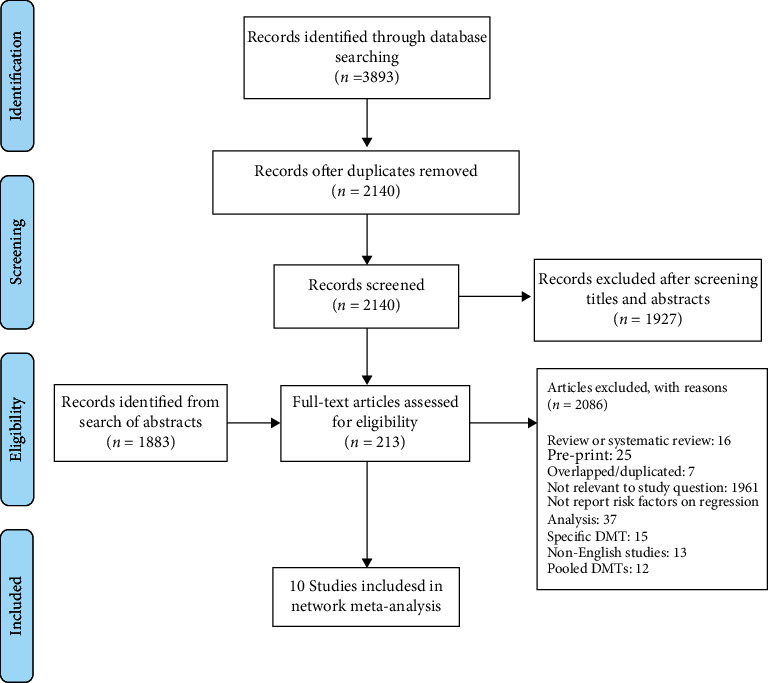
Study flow diagram.

A total of 3893 records were initially identified according to the research strategy. After duplicate removal, 2140 retrieved studies were screened in the title and abstract. Among 213 records reviewed in the full text, 10 published articles met inclusion criteria. Out of 1883 conference abstracts, none met inclusion criteria. Finally, a total of 10 studies were included in this systematic review. The PRISMA flow chart shows the process of study selection (Figure 1).

### 3.2. Characteristics of Studies Included

The characteristics of included studies are summarized in Tables 1(a) and 1(b). Five studies with 36912 MS patients consisting of 616 cases of suspected/confirmed COVID-19 investigated association of DMTs with COVID-19 susceptibility [9, 13–16]. Five studies, including 3639 MS patients with COVID-19, evaluated association of DMTs with COVID-19 severity [17–21]. Two of the included studies were cross-sectional [13, 17], 7 were cohort [9, 14–16, 18–21], and one was pharmacovigilance [9]. Four studies reported data from the USA [9, 16, 17, 21], two from Spain [15, 20], and one from each of Italy, [18] Iran, [13] and Sweden [19]. One study was multicentric from Europe [14].

The risk of bias judgment for each included study is presented in Supplementary Tables 1 and 2. Respecting the quality of cross-sectional studies, one was very good [17] and another one was unsatisfactory [13]. Regarding cohort studies, the qualities of 4 included studies were good and 4 were fair.

### 3.3. Network Meta-Analysis

#### 3.3.1. COVID-19 Susceptibility

**Figure 2:**
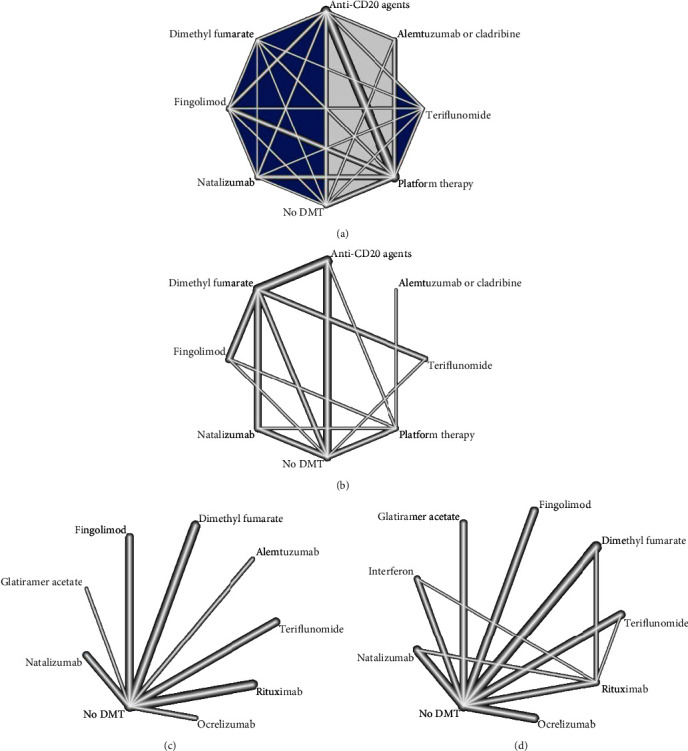
Network plots the effect of DMTs on the risk of acquiring COVID-19 and its severity. Platform therapy: interferon and glatiramer acetate; anti-CD20 agents: rituximab and ocrelizumab. (a) Risk of acquiring infection based on a univariate model. (b) Risk of acquiring infection based on a multivariate model. (c) Risk of severe infection based on a univariate model. (d) Risk of severe infection based on a multivariate model.

**Figure 3:**
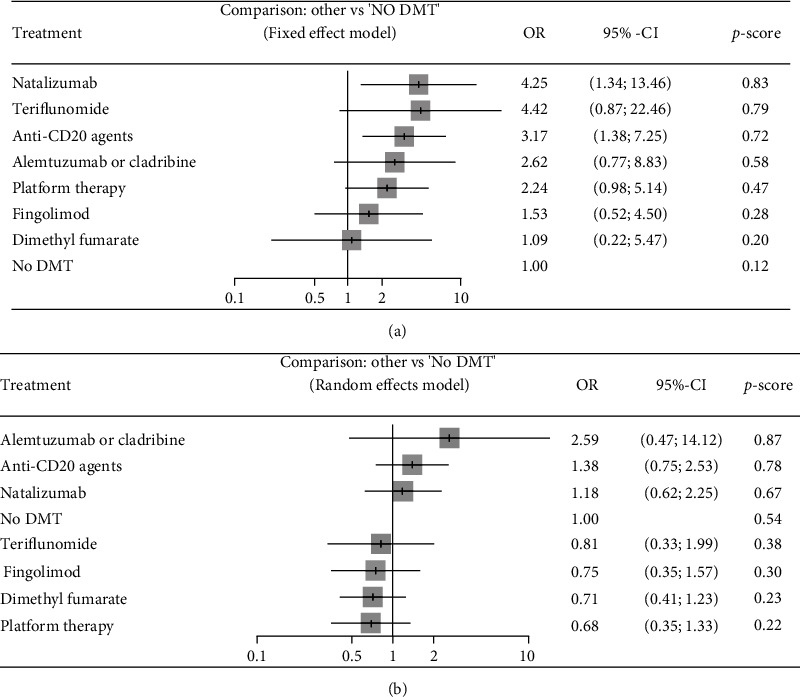
Forest plots of comparisons between DMTs and no DMTs for risk of acquiring COVID-19. Platform therapy: interferon and glatiramer acetate; anti-CD20 agents: rituximab and ocrelizumab. (a) Results of univariate analyses. (b) Results of multivariate analyses. *P* score ranges from zero to 1. A higher *P* score indicates a greater risk of being infected with COVID-19.

The network graphs and forest plots for the association of DMTs with the risk of acquiring COVID-19 are presented in Figures 2 and 3. Based on univariate analysis, three studies assessing the association of DMTs with the risk of COVID-19 were included in the NMA [13–15]. In comparison with no DMT, natalizumab (OR = 4.25, 95% CI: 1.34, 13.46; *P* score = 0.83) and anti-CD20 agents (OR = 3.17, 95% CI: 1.38, 7.25; *P* score = 0.72) were associated with higher risk of infection (Figure 3(a)). Ranking of the risk of infection identified dimethyl fumarate as the best, indicating lowest risk of developing infection, and natalizumab as the worst among DMTs. No significant results were found for other comparisons (Table 2(a)). There was a disagreement between direct and indirect comparison of no DMT with platform therapies (rituximab and glatiramer acetate). In direct comparison, no DMT was associated with a decreased risk of infection compared to platform therapies (OR = 0.39, 95% CI: 0.17, 0.92); but, no significant difference in indirect model was found (OR = 0.45, 95% CI: 0.19, 1.02). The total heterogeneity in NMA was not significant (*τ*^2^ = 0.072 and *I*^2^ = 19.4%, *Q*_total_ = 7.44, *P* = 0.282). There was no significant inconsistency between study designs (*Q*_between_ = 7.86, *P* = 0.249).

Three studies assessing the association between DMTs and risk of COVID-19 based on multivariate analysis were included in the NMA [9, 14, 16]. Pairwise comparisons showed that none of the DMTs had a worse effect on the risk of infection than another drug and no DMT (Table 2(b)). Ranking of the risk of infection identified interferon/glatiramer acetate as the best, indicating lowest risk, and alemtuzumab/cladribine as the worst among DMTs (Figure 3(b)). There was a disagreement between the direct and indirect comparisons of anti-CD20 agents with dimethyl fumarate. In direct comparison, the anti-CD20 agent's arm was associated with an increased risk of infection compared to dimethyl fumarate (OR = 3.25, 95% CI: 1.46, 7.24); but, we found no significant difference in the indirect model (OR = 1.88, 95% CI: 0.33, 10.73). We observed significant total heterogeneity (*τ*^2^ = 0.135 and *I*^2^ = 51.7%, *Q*_total_ = 14.48, *P* = 0.043). There was significant inconsistency between study designs (*Q*_between_ = 14.48, *P* = 0.043).

#### 3.3.2. COVID-19 Severity

**Figure 4:**
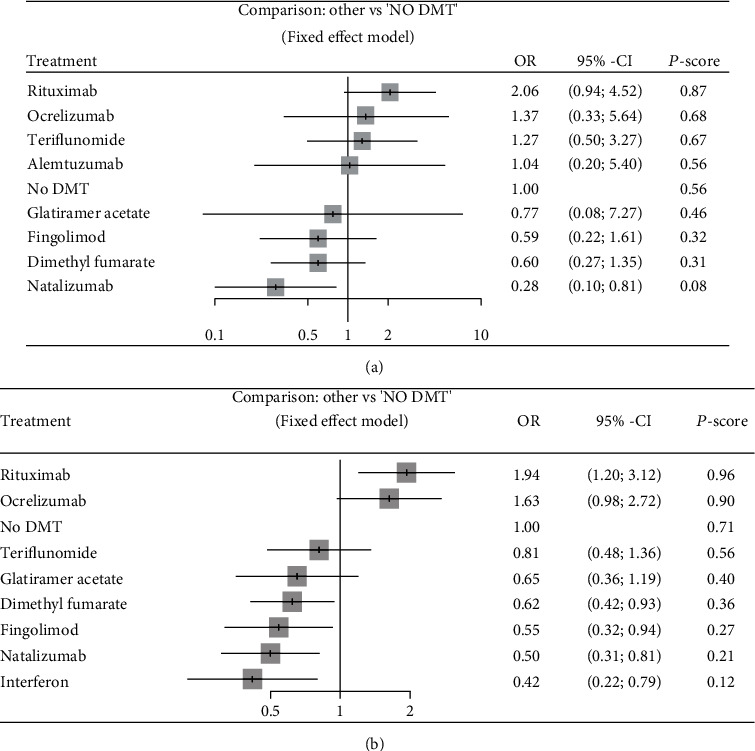
Forest plots of comparisons between DMTs and no DMTs for severity of COVID-19. (a) Results of univariate analyses. (b) Results of multivariate analyses. *P* score ranges from zero to 1. A higher *P*-score indicates a greater risk of developing severe COVID-19 infection.

The network graphs and forest plots for the association of DMTs with COVID-19 severity are presented in Figures 2 and 4. Based on univariate analysis, two studies assessing the association of DMTs with COVID-19 severity were included in the NMA [20, 21]. In the comparison of DMTs with no DMT, natalizumab was associated with a decreased risk of severe infection (OR = 0.28, 95% CI: 0.10, 0.81; *P* score = 0.08) (Figure 4(a)). For other comparisons, dimethyl fumarate (OR = 0.29, 95% CI: 0.09, 0.90) and natalizumab (OR = 0.14, 95% CI: 0.04, 0.51) were associated with lower risk of severe infection than rituximab (Table 3(a)). Additionally, natalizumab decreased the risk of severe infection (OR = 0.22, 95% CI: 0.05, 0.91) compared to teriflunomide. Ranking of the risk of severe infection identified natalizumab as the best and rituximab as the worst among DMTs. No significant total heterogeneity was detected (*τ*^2^ = 0.0 and *I*^2^ = 0%, *Q*_total_ = 2.06, *P* = 0.841). Additionally, there was no significant heterogeneity within designs (*Q*_within_ = 2.06, *P* = 0.841).

Four studies assessing the association of DMTs with COVID-19 severity based on multivariate analysis were included in the NMA [17–20]. In comparison with no DMT, dimethyl fumarate (OR = 0.62, 95% CI:0.42, 0.93; *P* score = 0.36), fingolimod (OR = 0.55, 95% CI:0.32, 0.94; *P* score = 0.27), natalizumab (OR = 0.50, 95% CI:0.31, 0.81; *P* score = 0.21), and interferon (OR = 0.42, 95% CI:0.22, 0.79; *P* score = 0.12) were associated with a decreased risk of developing severe COVID-19. However, rituximab increased the risk of severe infection compared to no DMT (OR = 1.94, 95% CI: 1.20, 3.12). Compared to rituximab and ocrelizumab, all DMTs were associated with a decreased risk of severe infection. Only the difference between teriflunomide and ocrelizumab was not significant. There were two disagreements between direct and indirect results for rituximab vs. teriflunomide and rituximab vs. interferon. Although rituximab was associated with an increased risk of severe disease compared to teriflunomide in the indirect model, no significant difference was found in the direct comparison (OR = 0.93, 95% CI: 0.19, 4.57). In the indirect comparison, interferon reduced the risk of severe infection compared to rituximab, but this reduction was not significant in the direct comparison (OR = 0.49, 95% CI: 0.10, 2.46). Ranking of the risk of severe infection identified interferon as the best and rituximab as the worst among DMTs (Figure 4(b)). No significant total heterogeneity was detected (*τ*^2^ = 0.139 and *I*^2^ = 0.373%, *Q*_total_ = 23.36, *P* = 0.077). There was significant heterogeneity within designs (*Q*_within_ = 19.93, *P* = 0.046), though no significant inconsistency was detected (*Q*_between_ = 3.43, *P* = 0.489).

## 4. Discussion

This study is aimed at summarizing the existing evidence on association of DMTs with COVID-19 susceptibility and severity in patients with MS. The finding of this network meta-analysis showed that patients on rituximab and ocrelizumab, and no DMT was at greater risk of severe COVID-19 infection compared to other MS patients. We observed no substantial difference across DMTs in the risk of developing severe infection.

When we ranked DMTs, interferon was associated with the lowest risk of acquiring COVID-19 and developing severe infection. This finding was also reported by Sormani et al. [22] that Italian MS patients on interferon were less likely to develop severe COVID-19 than those on other DMTs. These results were expected since interferon is not immunosuppressive and has anti-inflammatory and antiviral effects [23–26]. Protective effect of interferon against the SARS-CoV and MERS-CoV [27, 28], discovering autoantibodies against type I interferons in critically ill COVID-19 patients [29], and inhabitation effect of this agent on SARS-CoV-2 replication [30] suggested interferon as therapeutic candidate for COVID-19 [29, 31, 32]. However, the effectiveness of interferon on COVID-19 severity among general population in clinical trials remains unclear [33–35].

The harmful and beneficial effects of moderate and high effective DMTs on COVID-19 severity are still in dispute. Dimethyl fumarate, teriflunomide, and fingolimod decrease lymphocyte counts resulting in reduced viral clearance which may theoretically increase risk of severe COVID-19 infection [36–38]. Moreover, natalizumab limits viral clearance from the central nervous system [39] which could negatively affect the outcome of COVID-19 infection. However, experts and international recommendations suggested that these medications would not increase the risk of severe infection and may even have beneficial effects [40–42]. This network meta-analysis showed that none of the interferon, glatiramer acetate, dimethyl fumarate, teriflunomide, and natalizumab had a worse outcome compared to another one. All DMTs were also independently associated with a reduced risk of severe infection compared to no DMT, except anti-CD20 agents. This finding suggests that these medications are not likely to increase the risk of severe COVID-19 and are safe for using within the pandemic. Because of a lack of data, we could not examine the effect of alemtuzumab and cladribine on COVID-19 severity.

In the comparison of each specific DMT with no DMT, rituximab was associated with the highest risk of developing severe COVID-19 infection, followed by ocrelizumab. Observed increased risk of severe illness in patients treated with rituximab and ocrelizumab goes in line with studies on other autoimmune diseases [43–45]. Although the exact reason for this association is elusive, it is suggested that patients who treated with anti-CD20 monoclonal antibodies experience decreased antibody production, which can lead to an impaired immune response to SARS-CoV-2 [46–48]. Rituximab can also cause a decrease in CD4+ and CD8+ counts [49], which play a substantial role in response to SAR-CoV2 [50].

The results of primary studies showed a stronger association between rituximab and COVID-19 severity than ocrelizumab [17, 20, 22]. This difference could be related to the antibody-dependent cell-mediated cytotoxic effects and immunogenicity of these drugs [51, 52] or some confounders such as characteristics of patients and duration of treatment. The NMA on both univariate and multivariate results showed a decreased risk of developing severe COVID-19 in patients on ocrelizumab compared to rituximab. However, the differences were not substantially significant.

The NMA on univariate results identified lowest risk of developing COVID-19 in MS patients who received no DMT. However, the NMA on adjusted or multivariate results showed that platform therapies, fingolimod, dimethl fumarate, and teriflunomide had better outcome than no DMT. This inconsistency may be due the patients' characteristics. Most MS patients who received no DMTs are elderly and have advanced terminal stage. These patients are less involved in high-risk activities such as traveling, working outside the home, and spending a long time in social interaction. As a result, they may stay at home and not be in close contact with COVID-19 cases, which could decrease the risk of developing COVID-19.

One major issue in early research concerned the risk of acquiring COVID-19 in those who treated with anti-CD20 agents. Epidemiological and pharmacovigilance data suggested a higher risk of developing COVID-19 in MS patients on these agents [8, 9, 13]. However, some studies found no association between anti-CD20 medications and risk of the infection [14, 16, 53]. The suggested reasons for increased risk of acquiring infection in these agents are similar with those mentioned for increased risk of developing severe COVID-19. Although the pooled univariate results showed a higher risk of infection in patients treated with anti-CD20 agents than patients receiving no DMT, no notable difference between DMTs was detected after pooling multivariate analyses. These findings should be interpreted with caution since there was a high level of heterogeneity in NMA on multivariate analyses. Further work needs to be done to investigate the effect of DMTs on the risk of COVID-19 infection.

Our study has some limitations. First, we excluded non-English studies from the study. Second, there are differences in primary studies' health policies and medical care practices, which can affect our results. Third, we combined the quantitative findings of primary studies that used different adjustment methods. Fourth, the definition of COVID-19 susceptibility and severity varied among primary studies. Fifth, a limited number of studies included in quantitative analyses could dominate the estimates. Sixth, the primary used a different primary comparator (no therapy and no DMT). Seventh, this review is based on the current published articles, some of which were relatively small or did not have the necessary statistical power. Therefore, caution must be used when interpreting the association of DMTs with COVID-19 susceptibility or severity.

In conclusion, our study showed that MS patients on anti-CD20 agents are at greater risk of developing severe COVID-19 infection compared to those who received other DMTs and no DMT. It seems that other DMTs did not increase the risk of severe infection and are safe to continue during COVID-19 pandemic. We believed that our results are helpful to design appropriate programs to identify high-risk patients early and adapt vaccination strategies.

## Figures and Tables

**Table 1a:** (a) Characteristics of studies assessing association of DMTs with COVID-19 susceptibility

Author	Scenario of study	Type of study	Country reporting	Total MS patients	Number of suspected/confirmed COVID-19 cases	Definition of COVID-19 suspected or confirmed group	Analytical method used	Study quality
Sahraian et al., [30]	Contacted MS patients who were managed in the MS Clinic of Sina Hospital, Iran	Cross-sectional	Iran	4647	68	Patients were asked about COVID-19-related symptoms, CFT scan findings, PCR test, and hospitalization.	Univariate logistic regression	Unsatisfactory
Dalla Costa et al., [14]	A questionnaire sent to MS patients across Europe	Cohort	European multicentric	399	52	Patients experiencing fever or anosmia/ageusia+any other COVID-19 symptoms, or respiratory symptoms+two other COVID-19 Symptoms	Univariate and multivariate penalized likelihood logistic regression models	Good
Reder et al., [9]	Using the IBM Explorys real-world dataset	Pharmacovigilance	USA	30478	344	Patients with PCR-confirmed COVID-19 were considered COVID-19 positive; all others were considered COVID-19 negative.	Logistic regression adjusted for patient age, sex, BMI, comorbidities, and race	Good
Zabalza et al., [15]	Self-administered survey sent to patients were followed in Multiple Sclerosis Centre of Catalonia (Cemcat). Suspected COVID-19 cases were interviewed by phone.	Cohort	Spain	758	48	(1) Patients with fever, dyspnoea, persistent cough, or (2) sudden onset of anosmia, ageusia or dysgeusia, or (3) radiological images compatible with COVID-19 were considered suspected cases. Patients with a positive SARS-CoV-2 PCR were considered confirmed cases	Univariable and multivariable logistic regressions	Good
Levin et al., [16]	Online surveys using the Research Electronic Data Capture (REDCap) platform was sent to patients MS or a related disorder across USA	Cohort	USA	630	104	(1) Patients with cough or shortness of breath, or (2) any two of the following: fever, muscle pain, sore throat, and new loss of taste or smell	Multivariate logistic regressions	Fair

**Table 1b:** (b) Characteristics of studies assessing association of DMTs with COVID-19 severity

Author	Scenario of study	Type of study	Country reporting	Total MS patients with COVID-19	Number of severe cases	Definition of COVID-19 severity	Analytical method used	Study quality
Salter et al., [17]	Registry of MS and patients with confirmed or suspected COVID-19 in North America (COViMS Registry)	Cross-sectional	North America	1626	333∗	(a) Requiring hospitalization only (b) ICU and/or required ventilator support (c) Death	Multivariable multinomial logistic regression	Very good
Sormani et al., [18]	Collected data of MS patients who had been in contact with their neurologist because of a confirmed or suspected COVID-19 (MUSC-19 registry)	Cohort	Italy	844	136	(a) No need for hospitalization or documented diagnosis of pneumonia (b) Diagnosis of pneumonia or hospitalization (c) Death or ICU admission	Univariate and multivariate ordinal logistic regressions	Fair
Spelman et al., [19]	Registry of Swedish MS patients with suspected and confirmed COVID-19 infection (SMSreg)	Cohort	Sweden	476	73	(a) Not requiring hospitalization (b) Hospitalization, ICU, or death	Weighted logistic regression with IPTW approach to adjust confounders	Fair
Moreno-Torres et al., [20]	Registry of MS and patients with confirmed or highly suspected COVID-19 across Madrid	Cohort	Spain	219	51	(a) No need for hospitalization (b) Requiring hospitalization	Univariate and multivariate logistic regression models with an L1 penalty (Lasso regression)	Good
Klineova et al., [21]	Patients with MS or related CNS disorders with suspected or confirmed COVID-19 in New York or surrounded city (NYCNIC registry)	Cohort	USA	474	58	(a) Not requiring hospitalization (b) Hospitalization, ICU, or death	Univariable and multivariable logistic regressions	Fair

^∗^Only hospitalized patients. ICU: intensive care unit.

**Table 2a:** (a) Results from univariate analyses

**ALZ or CLA**	0.27 (0.06, 1.28)	.	.	.	1.29 (0.24, 6.90)	1.07 (0.41, 2.80)	.
0.83 (0.30, 2.27)	**Anti-CD20 agents**	3.11 (0.75, 12.94)	2.56 (0.91, 7.20)	0.70 (0.17, 2.98)	**2.86** **(1.21, 6.77)**	1.32 (0.84, 2.07)	0.77 (0.18, 3.24)
2.40 (0.43, 13.3)	2.90 (0.70, 12.03)	**DMF**	0.82 (0.15, 4.52)	0.23 (0.03, 1.64)	1.33 (0.19, 9.47)	0.55 (0.13, 2.43)	0.25 (0.03, 1.78)
1.71 (0.51, 5.69)	2.07 (0.94, 4.54)	0.71 (0.15, 3.45)	**FNG**	0.28 (0.05, 1.53)	1.61 (0.29, 8.84)	0.74 (0.34, 1.60)	0.30 (0.05, 1.66)
0.62 (0.17, 2.17)	0.74 (0.31, 1.82)	0.26 (0.05, 1.32)	0.36 (0.12, 1.08)	**NTZ**	5.85 (0.81, 42.25)	1.97 (0.85, 4.56)	1.09 (0.15, 7.94)
2.62 (0.77, 8.83)	**3.17** **(1.38, 7.25)**	1.09 (0.22, 5.47)	1.53 (0.52, 4.50)	**4.25** **(1.34, 13.46)**	**No DMT**	**0.39** **(0.17, 0.92)**	0.19 (0.03, 1.34)
1.17 (0.45, 3.02)	1.41 (0.92, 2.17)	0.49 (0.11, 2.06)	0.68 (0.32, 1.45)	1.90 (0.82, 4.38)	0.45 (0.19, 1.02)	**Platform therapy**	0.45 (0.10, 1.99)
0.59 (0.11, 3.33)	0.72 (0.17, 3.01)	0.25 (0.03, 1.78)	0.35 (0.07, 1.70)	0.96 (0.19, 5.00)	0.23 (0.04, 1.15)	0.51 (0.12, 2.18)	**TRF**

**Table 2b:** (b) Results from multivariate analyses

**ALZ or CLA**	.	.	.	.	.	3.78 (0.79, 18.00)	.
1.88 (0.33, 10.73)	**Anti-CD20 agents**	**3.25** **(1.46, 7.24)**	.	.	0.91 (0.39, 2.12)	1.27 (0.31, 5.13)	.
3.64 (0.65, 20.46)	1.88 (0.33, 10.73)	**DMF**	0.87 (0.37, 2.05)	0.71 (0.30, 1.69)	1.07 (0.40, 2.85)	.	1.12 (0.44, 2.89)
3.48 (0.59, 20.33)	3.64 (0.65, 20.46)	0.96 (0.48, 1.90)	**FNG**	.	0.68 (0.16, 2.82)	0.94 (0.25, 3.57)	.
2.19 (0.38, 12.49)	3.48 (0.59, 20.33)	0.60 (0.32, 1.14)	0.63 (0.27, 1.49)	**NTZ**	1.27 (0.51, 3.19)	2.25 (0.60, 8.42)	.
2.59 (0.47, 14.12)	2.19 (0.38, 12.49)	0.71 (0.41, 1.23)	0.75 (0.35, 1.57)	1.18 (0.62, 2.25)	**No DMT**	1.69 (0.66, 4.38)	0.65 (0.15, 2.92)
3.78 (0.79, 18.00)	2.59 (0.47, 14.12)	1.04 (0.50, 2.18)	1.09 (0.48, 2.49)	1.73 (0.80, 3.76)	1.46 (0.75, 2.84)	**Platform therapy**	.
3.18 (0.49, 20.81)	3.78 (0.79, 18.00)	0.87 (0.39, 1.97)	0.91 (0.32, 2.58)	1.46 (0.54, 3.94)	1.23 (0.50, 2.99)	0.84 (0.30, 2.39)	**TRF**

On the upper triangle, the effect size are direct comparisons; the effect sizes presented on lower triangle are network meta-analyses (indirect comparison). Comparisons should be read from left to right (example for upper triangle: OR (95% CI) of developing COVID-19 in anti-CD20 agents compared to DMF is 3.25 (1.46, 7.24); example for lower triangle: OR (95% CI) of developing COVID-19 in the ALZ or CLA group compared to anti-CD20 agents is 1.88 (0.33, 10.73). Platform therapy: interferon and glatiramer acetate; anti-CD20 agents: rituximab and ocrelizumab. ALZ: alemtuzumab; CLA: cladribine; DMF: dimethyl fumarate; FNG: fingolimod; NTZ: natalizumab; TRF: teriflunomide; DMT: disease-modifying therapy.

**Table 3a:** (a) Results from univariate analyses

**ALZ**	.	.	.	.	1.04 (0.20, 5.40)	.	.	.
1.74 (0.28, 10.92)	**DMF**	.	.	.	0.60 (0.27, 1.35)	.	.	.
1.75 (0.26, 12.01)	1.01 (0.28, 3.65)	**FNG**	.	.	0.59 (0.22, 1.61)	.	.	.
1.35 (0.08, 21.89)	0.78 (0.07, 8.47)	0.77 (0.07, 9.01)	**GA**	.	0.77 (0.08, 7.27)	.	.	.
3.65 (0.52, 25.72)	2.10 (0.56, 7.91)	2.09 (0.49, 8.85)	2.71 (0.23, 32.21)	**NTZ**	**0.28** **(0.10, 0.81)**	.	.	.
1.04 (0.20, 5.40)	0.60 (0.27, 1.35)	0.59 (0.22, 1.61)	0.77 (0.08, 7.27)	**0.28** **(0.10, 0.81)**	**No DMT**	0.73 (0.18, 3.01)	0.49 (0.22, 1.07)	0.78 (0.31, 2.02)
0.76 (0.09, 6.67)	0.44 (0.09, 2.24)	0.43 (0.08, 2.45)	0.56 (0.04, 7.99)	0.21 (0.04, 1.21)	0.73 (0.18, 3.01)	**OCR**	.	.
0.51 (0.08, 3.14)	**0.29** **(0.09, 0.90)**	0.29 (0.08, 1.03)	0.37 (0.03, 4.04)	**0.14** **(0.04, 0.51)**	0.49 (0.22, 1.07)	0.67 (0.13, 3.36)	**RTX**	.
0.82 (0.12, 5.45)	0.47 (0.14, 1.63)	0.47 (0.12, 1.84)	0.60 (0.05, 6.91)	**0.22** **(0.05, 0.91)**	0.78 (0.31, 2.02)	1.08 (0.20, 5.89)	1.62 (0.47, 5.52)	**TRF**

**Table 3b:** (b) Results from multivariate analyses

**DMF**	.	.	.	.	**0.62** **(0.40, 0.95)**	.	**0.34** **(0.12, 0.98)**	.
1.14 (0.58, 2.22)	**FNG**	.	.	.	**0.55** **(0.32, 0.94)**	.	.	.
0.95 (0.46, 1.96)	0.84 (0.38, 1.87)	**GA**	.	.	0.65 (0.36, 1.19)	.	.	.
1.49 (0.70, 3.14)	1.31 (0.57, 3.01)	1.56 (0.65, 3.74)	**IFN**	.	**0.36** **(0.18, 0.72)**	.	0.49 (0.10, 2.46)	.
1.24 (0.67, 2.30)	1.09 (0.53, 2.24)	1.30 (0.61, 2.80)	0.83 (0.38, 1.84)	**NTZ**	**0.51** **(0.31, 0.86)**	.	**0.23** **(0.07, 0.78)**	.
**0.62** **(0.42, 0.93)**	**0.55** **(0.32, 0.94)**	0.65 (0.36, 1.19)	**0.42** **(0.22, 0.79)**	**0.50** **(0.31, 0.81)**	**No DMT**	0.61 (0.37, 1.02)	**0.39** **(0.20, 0.75)**	1.37 (0.80, 2.36)
**0.38** **(0.20, 0.73)**	**0.34** **(0.16, 0.70)**	**0.40** **(0.18, 0.88)**	**0.26** **(0.11, 0.58)**	**0.31** **(0.15, 0.62)**	0.61 (0.37, 1.02)	**OCR**	.	.
**0.32** **(0.18, 0.57)**	**0.28** **(0.14, 0.58)**	**0.34** **(0.16, 0.73)**	**0.22** **(0.10, 0.46)**	**0.26** **(0.14, 0.48)**	**0.52** **(0.32, 0.83)**	0.84 (0.42, 1.69)	**RTX**	0.93 (0.19, 4.57)
0.77 (0.40, 1.47)	0.67 (0.32, 1.42)	0.80 (0.37, 1.77)	0.52 (0.23, 1.17)	0.62 (0.31, 1.24)	1.23 (0.74, 2.06)	2.01 (0.97, 4.15)	**2.39** **(1.22, 4.66)**	**TRF**

On the upper triangle, the effect size are direct comparisons; the effect sizes presented on lower triangle are network meta-analyses (indirect comparison). Comparisons should be read from left to right (example for upper triangle: OR (95% CI) of developing a severe COVID-19 in DMF compared to no DMT is 0.62 (0.40, 0.95); example for lower triangle: OR (95% CI) of developing a severe COVID-19 in DMF compared to FNG is 1.14 (0.58, 2.22). DMF: dimethyl fumarate; FNG: fingolimod; GA: glatiramer acetate; IFN: interferon; NTZ: natalizumab; TRF: teriflunomide; DMT: disease-modifying therapy; RTX: rituximab; OCR: ocrelizumab.

## Data Availability

All supporting the results of this study can be found within the tables, figures, and manuscript of the present study.

## Supplementary Materials

Supplementary Materials Table S1: quality assessment of the included cross-sectional studies based on the NOS checklist. Table S2: quality assessment of the included cohort studies based on the NOS checklist. Pages 3-5: PRISMA 2020 checklist.
